# Energy consumption assessment in manufacturing Ti6Al4V electron beam melted parts post-processed by machining

**DOI:** 10.1007/s00170-022-10794-z

**Published:** 2023-01-06

**Authors:** Ersilia Cozzolino, Stefania Franchitti, Rosario Borrelli, Carmine Pirozzi, Antonello Astarita

**Affiliations:** 1 Department of Chemical, Materials and Production Engineering, University of Naples “Federico II”, P.Le Tecchio 80, 80125 Naples, Italy; 2 CIRA (Italian Aerospace Research Center), 81043 Capua, CE Italy

**Keywords:** Titanium alloys, Sustainability, Turning, Electron beam melting, Energy consumption, Additive manufacturing

## Abstract

The assessment of energy consumed in manufacturing operations and the enhancement of their sustainability plays a fundamental role in the present research contest. Electron beam melting (EBM) is an additive manufacturing technique that allows the fabrication of titanium parts with high productivity and a low buy-to-fly ratio; on the other hand, the roughness of the parts is not adequate for high-performance applications, so a finishing step is always required. Aiming to reduce the energy used to produce a part, all the required manufacturing steps should by carefully treated in an integrated framework. The aim of this paper is to study the energy required to produce a Ti6Al4V part printed through EBM and the machined to achieve the desired surface finishing. Cylindrical specimens have been printed through an Arcam machine by using the processing conditions suggested by the manufacturer; then, the specimens have been turned under different processing conditions. The energy required in all the phases has been recorded and then carefully analyzed to point out the processing conditions which allows a more efficient use of resources. The results showed that the printing phase is by far the most energy demanding so should be carefully treated to reduce the printing time even if a greater roughness is achieved; the analysis of the machining stage suggested that both depth of cut and spindle speed must be kept the higher as possible to reduce the energy consumption of this stage.

## Introduction

In the current scenario of sustainable development, sustainable manufacturing (SM) is becoming the main goal to achieve and pursue for both the research community and industry. An excellent diversity of interpretations related to SM exist [1]. In general, it can be considered as a triple-bottom-line concept including three main pillars to be satisfied, which are as follows: environment, economy, and society. In this perspective, manufacturing by pursuing a sustainable approach not only means providing economic satisfaction but also meeting social and environmental standards. Actually, manufacturing has always been seen as the key factor for the prosperity and development of countries [2] because it transforms materials into goods for satisfying human requirements. Nevertheless, they are a significant source of environmental pollution in terms of energy consumption, material waste, and CO_2_ emissions.

Additive manufacturing (AM) plays a crucial role in the ongoing industrial context. It consists of the manufacturing of three-dimensional objects by adding layers of material based on a three-dimensional computer model. First and foremost, AM is currently becoming a key technology in industries such as aerospace, biomedicine, and manufacturing as it allows the fabrication of customized, novel, and complex structures, such as free form enclosed structures and channels and lattices can be achievable [3]. Then, the direct production from the 3D CAD models results in no tools and moulds are required to realize the parts, so there is no switch over costs. It also has allowed to deal with unforeseen events such as COVID-19 pandemic with the production of face masks, test swabs, door handle attachments, valves, and face shields [4].

Electron beam melting (EBM) is among the most widely used AM techniques to additively build titanium parts layer by layer for lots of applications, such as biomedical, automotive, and aerospace [5]. The EBM process is carried out in a vacuum at an elevated temperature to obtain stress-relaxed objects having mechanical and material properties better than wrought forging or cast. Nevertheless, surface finishing of EBM parts hardly ever meets industrial quality requirements so post-process machining is often needed. Thus, post-treatment is always required so that the additively manufactured parts reach a better surface finishing. According to the literature, heat treatments [5, 6], electrochemical polishing [7], laser polishing [8], and machining [9] are among the most employed post-treatments to improve the surface roughness of EBM parts.

AM is claimed to be a green technology because of its materials efficiency resulting in time and cost reduction for manufacturing small-volume parts when compared to conventional technologies [10]. Some studies exist in the literature on the sustainability assessment of additive manufacturing processes. Kellens et al. [11] investigated the environmental impact of the selective laser sintering process [12], by adopting life cycle inventory (LCI) data from CO2PE!, a methodology evaluating energy, resources, and process emissions of manufacturing processes. They found that process time and resource consumption were correlated to process parameters such as laser power, build volume, printing speed, and layer thickness. Peng et al. [13] investigated the influence of process parameters on quality and energy consumption to manufacture AlSi10Mg parts by selective laser melting (SLM) process. Specific energy consumption (SEC) was adopted as an efficiency index to estimate the sustainability of the process. Their results showed that electrical energy can be saved while satisfying quality standards by appropriately selecting the process parameters in the SLM process. Lunetto et al. [14] investigated the relationship between the specific energy consumption (SEC) and the deposition rate for the EBM process by considering different manufacturing designs. They found a hyperbolic law between time and energy efficiency due to the process control of the machine.

Liu et al. [15] compared the energy consumption of different AM processes, and they found that the EBM has the lowest energy consumption (60 MJ/kg) because of its high process rate whereas the direct energy deposition (DED) process has the highest energy consumption (7708 MJ/kg). Baumers et al. [16] investigated the effect of the variation in shape complexity of a part on energy consumption during the EBM process. They found a weak correlation between them by concluding that energy consumption is not driven by shape complexity.

Sustainable machining has also been investigated in the literature. Kurukulasuriya et al. [17] collected the energy consumption data using a data logger and then quantified the environmental impact associated with the milling process by means of a Life Cycle Assessment (LCA) software which uses ISO 14044 guidelines. Their results showed that the workpiece material as well as the electrical energy contribute mostly to the adverse impact on the environment. Khanna et al. [18] compared the sustainability performance of the conventional flood coolant with minimum quantity lubrication (MQL) and liquid carbon dioxide as a cryogenic coolant in machining Ti6Al4V. They found that MQL machining has a lower impact on the environment, but it is not sustainable because it led to a 75% reduction of tool life with a higher cutting force and surface roughness. Flood machining was also found to be non-sustainable while cryogenic machining showed to have a good balance between machining and sustainable performances. Indeed, cryogenic machining is considered a candidate for sustainable coolant application [19].

Also, the concept of hybrid manufacturing, combining additive, and subtractive technologies is becoming more and more important today to provide solutions to both global market requirements and industrial needs. It is designed as a potential solution for manufacturing and repairing operations as it leads to material waste reduction, time consumption, and flexibility. However, it faces some challenges such as digitalization and process parameterization, CAD/CAM solutions. Gonzàlez-Barrio et al. [20] proposed a new methodology for a hybrid manufacturing system. Concretely, they realized a programmed interface to interact between laser metal deposition and milling, by including all hybrid stages inside the same environment and facilitating the hybrid process manufacturing. The results of this study demonstrate the necessity of consistent knowledge to guarantee the interaction between different technologies.

Hence, some studies investigated the energy consumption of AM processes, and many works are focused on the energy consumption of machining processes. Nevertheless, very few studies investigate the energy consumption of both AM and post-process machining. It is a gap of knowledge that this study aims to fill according to the fact that, as previously mentioned, AM parts always require post-processing to meet the quality standard or to be assembled. From a sustainable perspective, the additional steps to manufacturing processes unavoidably result in additional energy demand and resources, and this cannot be neglected. Moreover, considering the integration of both AM and post-process, as often is done for practical industrial applications, could lead to a decrease in the sustainable advantages of the AM. These reasons prompted this experimental study, whose aim is to understand how to obtain Ti6Al4V parts manufactured by EBM and then machined by turning by saving energy without sacrificing the roughness. The titanium alloy Ti6Al4V is the material used in this experimental campaign as it is among the most used in AM due to its good material properties, such as corrosion resistance, high strength, and biocompatibility [21].

Ti6Al4V samples were, firstly, manufactured by means of an EBM machine while working in automatic mode. Then, all the samples were machined by means of a lathe removing by varying the process parameters to remove a total depth of cut of 3 mm. The roughness of the samples was measured both before and after the machining process as well as the electrical energy was acquired during both the EBM and turning process to correlate the sustainability of the process in terms of energy consumption with the reached roughness. The choice of keeping constant the total depth of cut (3 mm) derives from the objective of making measurements and then the results in terms of both roughness and energy consumption comparable. In other words, this study aims to comprehend how to properly select the process parameters to minimize energy consumption during the turning process of EBMed Ti6Al4V parts. Thus, by fixing the process parameters in the EBM process and the surface finishing to be obtained in terms of surface roughness, the results of this work aim to provide guidelines in selecting turning process parameters to obtain Ti6Al4V parts having the desired surface finishing by minimizing energy consumption.

As the existing literature lacks studies on the sustainability of AM and post-process processing jointly evaluated [3], the results obtained in this study could find immediate practical application to improve the industrial sustainability of production processes. Some guidelines will be released on how to select the best cut strategy in post-process turning to obtain a good surface finishing of EBMed Ti6Al4V parts by minimizing energy consumption. The integration of both AM and post-process machining demonstrates that environmental performance needs to be considered both in the design and production planning stages of AM processes to make long-term sustainable decisions.

## Material and methods

**Fig. 1:**
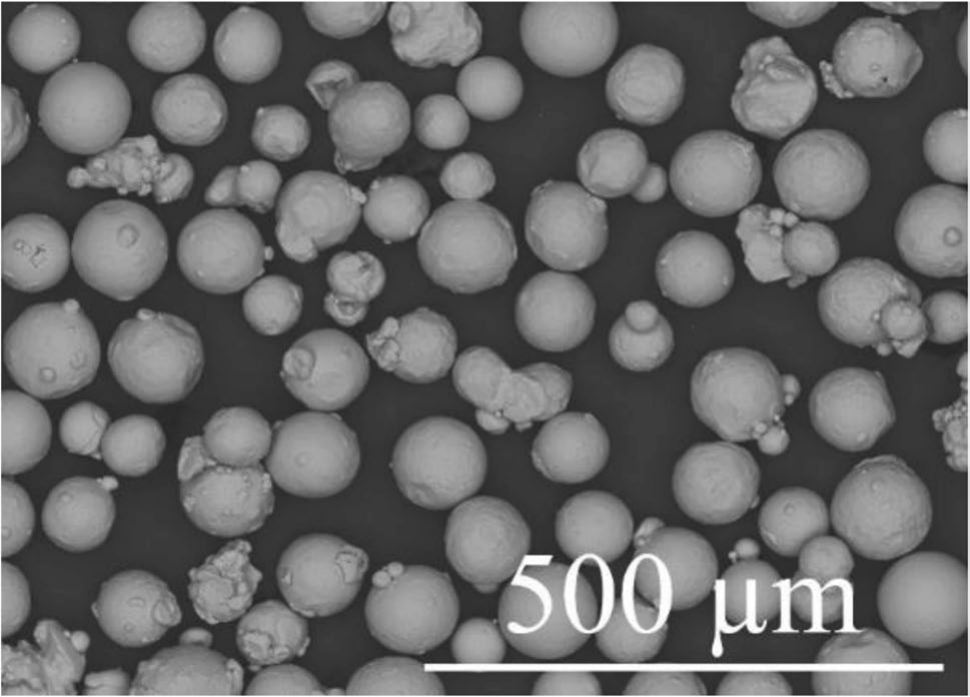
SEM image of the Ti6Al4V powders used (magnification × 200)

Plasma-atomized-virgin powders of Ti6Al4V with a particle size going from 45 to 106 µm and an apparent density of 2.57 g/cm^3^ [22] were used in this study. A scanning electron microscope (SEM) image of the powders is given in Fig. 1 while the chemical composition is reported in Table 1.

**Table 1:** Chemical composition of the powders used

Elements	Al	V	Fe	O	N	H	C	Ti
Wt%	6.40	4.12	0.18	0.14	0.01	0.003	0.01	Balance

An Arcam A2X machine with a build volume of 200 × 200 × 380 mm^3^ and operating under vacuum (10^−4^–10^−5^ mbar) was adopted to print the samples. The electrons were emitted by a hot tungsten filament cathode, accelerated at 60 kV, and focused and deflected by means of electromagnetic lenses to perform the EBM process. The machine worked in automatic mode so that an intrinsic algorithm delineated the beam current and the beam speed.

The samples were manufactured by using the standard Ti6Al4V build theme suggested by Arcam [23], and a helium pressure of 10^−3^ mbar was applied to control the vacuum and avoid powder jumping, due to electrostatic charging or smoke events, which can lead to stopping the process [24]. Each layer was built in four steps: powder spreading, preheating, melting, and platform lowering [14]. Layer thickness *t* and the line offset $h$ were fixed for all the samples equal to 50 µm and 0.1 mm, respectively, while the electron beam parameters were varied throughout the build as specified by the inner algorithm of the machine to obtain fully dense parts having consistent properties and microstructure [23, 25].

Thus, the Ti6Al4V cylindrical samples had been printed under the same process conditions, and then, they were post-processed under different turning conditions in this study.

The microstructure of the printed samples has been observed by means of a Hitachi TM3000 SEM; the specimens for metallographic observations have been cut from the samples by means of a metallographic precision hacksaw; then, the specimens have been hot mounted in conductive resin. The specimens are taken in the cross-section of the samples; perpendicular to the building direction, two different areas have been observed: the core of the samples and the outer region. The specimens have been then polished to a mirror-like surface finishing; lastly, a chemical etching (using the Kroll solution) has been performed to reveal the microstructure.

Adria Machine FEL-660HG lathe was adopted to perform the post-process machining. Three levels of spindle speed *N* (440 rev/min, 680 rev/min, and 1200 rev/min) and depth of cut *a* (0.5 mm, 1 mm, and 1.5 mm) were respectively adopted to perform the turning process. The feed rate *f* was fixed equal to 0.22 mm/rev, and the total depth of cut was chosen equal to 3 mm for all the samples to compare the energy required to remove a given amount of material. Spindle speed, cutting speed, depth of cut and, then, the number of cutting passes were varied to achieve the same total depth of cut (3 mm) (Table 2). Thus, all the process conditions adopted in this study have been chosen by taking into account the best results in terms of surface roughness in the literature [26, 27] that could meet the industrial quality requirements for Ti6Al4V parts.

**Table 2:** The combinations of process parameters adopted in turning process

Combination	*N* (rev/min)	*a* (mm)	Number of cutting passes	Total depth of cut (mm)	${{{V}}}_{{{c}}}$(m/min)	MRR (mm^3^/s)
1	440	0.5	6	3	42	77
2	440	1	3	3	42	154
3	440	1.5	2	3	42	231
4	680	0.5	6	3	64	117
5	680	1	3	3	64	235
6	680	1.5	2	3	64	352
7	1120	0.5	6	3	106	194
8	1120	1	3	3	106	389
9	1120	1.5	2	3	106	583

The cutting speed ${V}_{c}$ was calculated for each combination of process condition as [28]:

1 $${V}_{c}=\pi *D*N /1000$$

where $D$ is the diameter of the samples, equal to 30 mm, and $N$ is the spindle speed. 60° triangular inserts Sandvik Coromant were used as cutting tools to perform the machining process and Siroil Emulg 3/10 was adopted as lubricant oil to prevent overheating and wear. The material removal rate (MRR) is a performance index which describes the amount of material removed per time unit. It represents a productivity index and is calculated as follows:

2 $$\mathrm{MRR}=f*a *{V}_{c}$$

where $f$ is the feed rate, $a$ is the dept of cut and ${V}_{c}$ is the cutting speed.

The energy consumption during both the processes under investigation, electron beam melting and turning, was measured and recorded by means of a Qualistar Plus Power and Energy Quality Analyser CA8331, that is a commercial system. The device is equipped with current sensor and tension cables with crocodile clips. It uses the absolute measurements of the current and tension to measure the power and energy consumption over time.

**Fig. 2:**
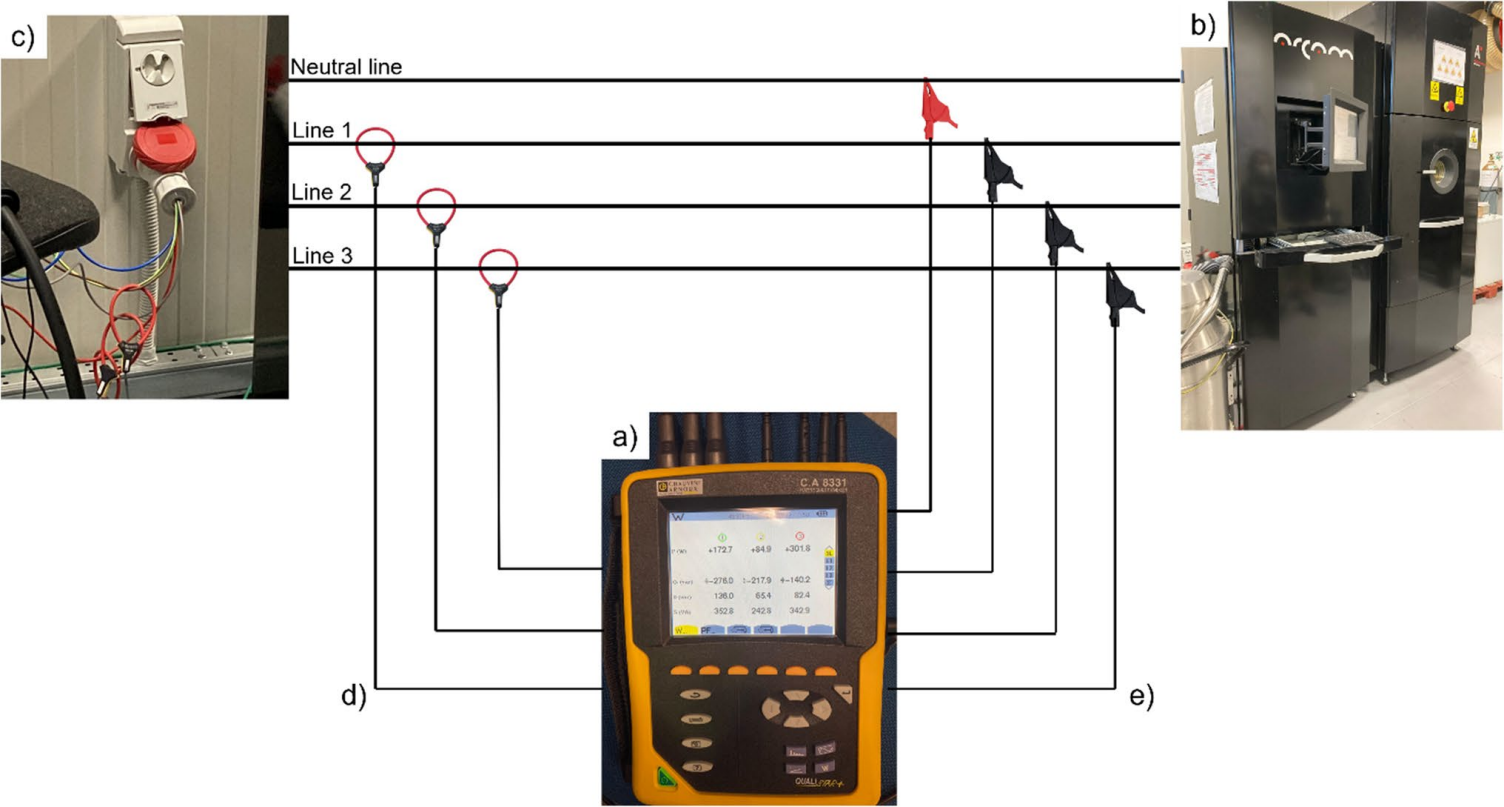
Experimental setup of the EBM process: (**a**) energy device, (**b**) EBM machine, (**c**) electric plant, (**d**) current sensors, and (**e**) tension cables

The EBM machine has a three-phase connection with neutral 32 A 400 V so three current sensors MiniFLEX MA193-350 and four tension cables with 4 crocodile clips were used for the acquisitions, as shown in Fig. 2, by fixing a sampling period of one second.

**Fig. 3:**
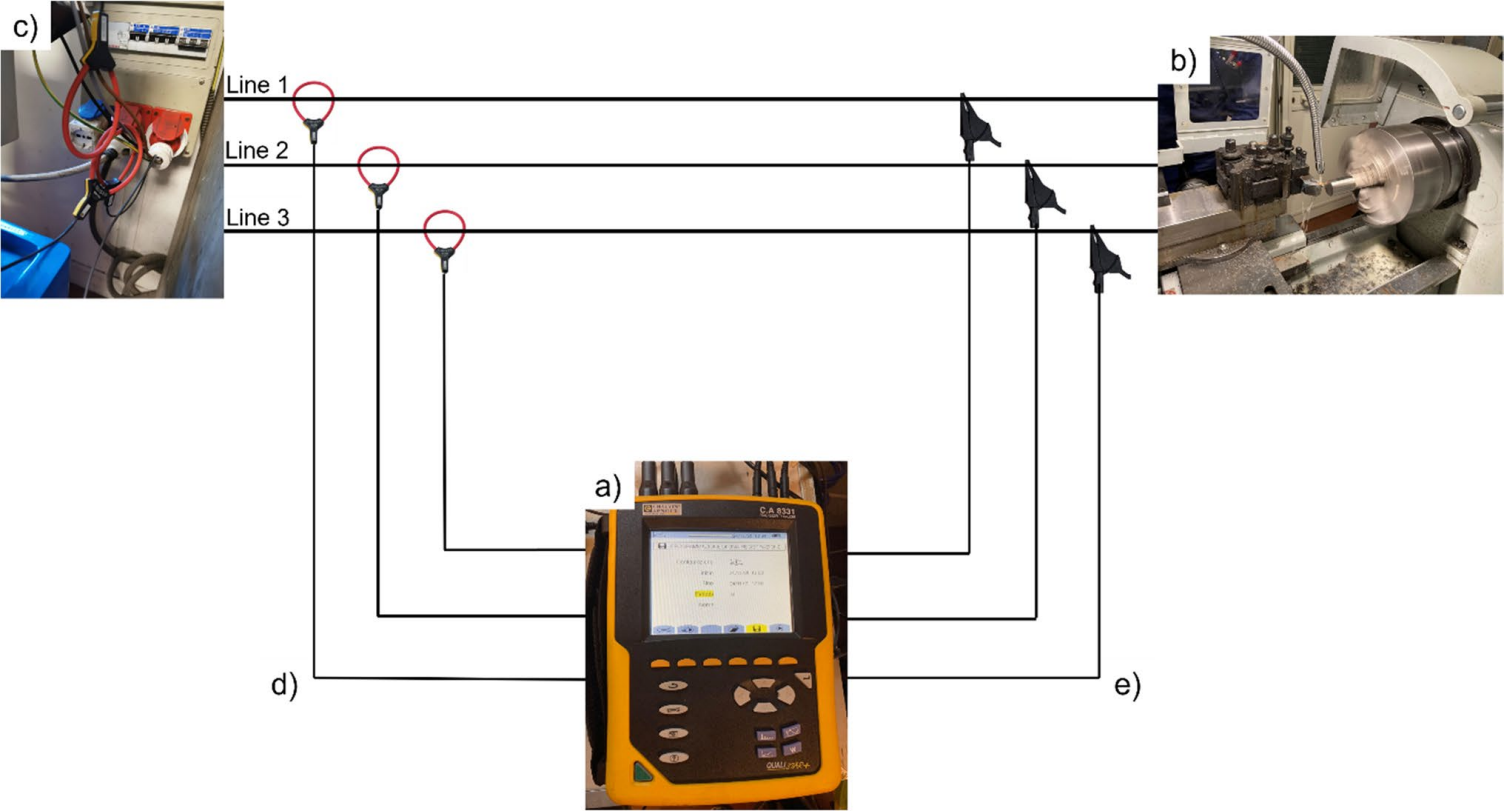
Experimental setup of the turning process: (**a**) energy device, (**b**) lathe, (**c**) electrical plant, (**d**) current sensors, and (**e**) tension cables

The lathe has got a three-phase connection without neutral 32 A 380 V so three current sensors and only three tension cables with three crocodile clips were adopted as illustrated by the scheme in Fig. 3. The sampling period was chosen equal to one second for data acquisition.

The surface roughness of the samples was acquired both before and after the turning process by means of the confocal microscope 3D Optical Surface Metrology System Leica DCM3D. The roughness was measured, according to EN ISO 4287 standard, along two directions: (i) along the direction parallel to the axis of the cylindrical samples before and after the turning process by choosing an evaluation length of 12.5 mm and a cut-off length of 2.5 mm and (ii) along the perpendicular direction an evaluation length of 4 mm and a cut-off length of 0.8 mm were chosen for the measurements. Ra parameter, calculated as defined by the above-mentioned standard, was chosen as studied output. The purpose of these measurements is to define the less energy demanding energy conditions ensuring, at the same time, the desired surface finishing.

## Results

**Fig. 4:**
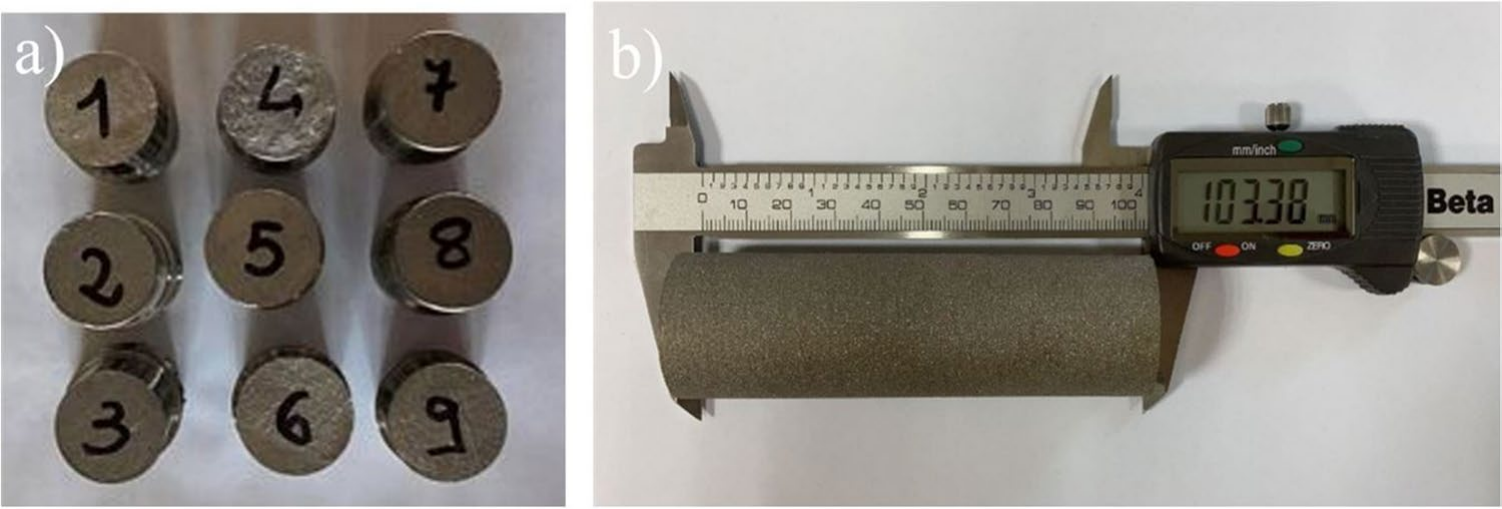
The as-built EBM samples: (**a**) all the nine cylindrical samples and (**b**) the sample number 1

The printed samples used in this experimental campaign are shown in Fig. 4; as expected, all the samples were correctly printed and did not show any appreciable defect.

**Fig. 5:**
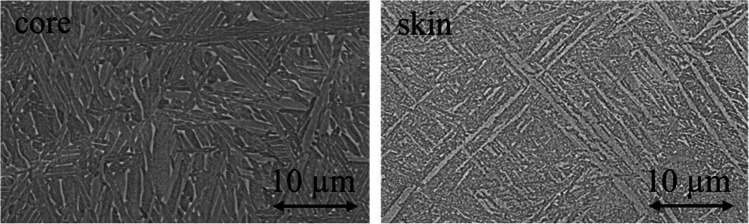
Cross-section SEM micrographs of the samples, representative of both the core and the outer layer of the samples as printed

In Fig. 5 cross-section, SEM micrographs representative for all the samples are given. The same microstructure has been observed for all of them as they have been printed under the same processing conditions and by using the parameters suggested by Arcam.

In agreement with the available literature, a basket-weave Widmanstätten-like microstructure is observed; this structure is the typical one achieved from the fast solidification and consequent cooling down of molten titanium [29]. The width of the lamellae is linked to the cooling rate; the higher is the cooling rate, the thinner are the lamellae; the dimensions of the lamellae reflect also the hardness of the material: the thinner the lamellae, the harder the alloy [30]. The outer region of the sample solidified under a higher cooling rate resulting in a harder structure; this point is of interest because this outer layer is the one removed by machining, so a harder machining process can be expected due to the hardness of the material in this region.

**Fig. 6:**
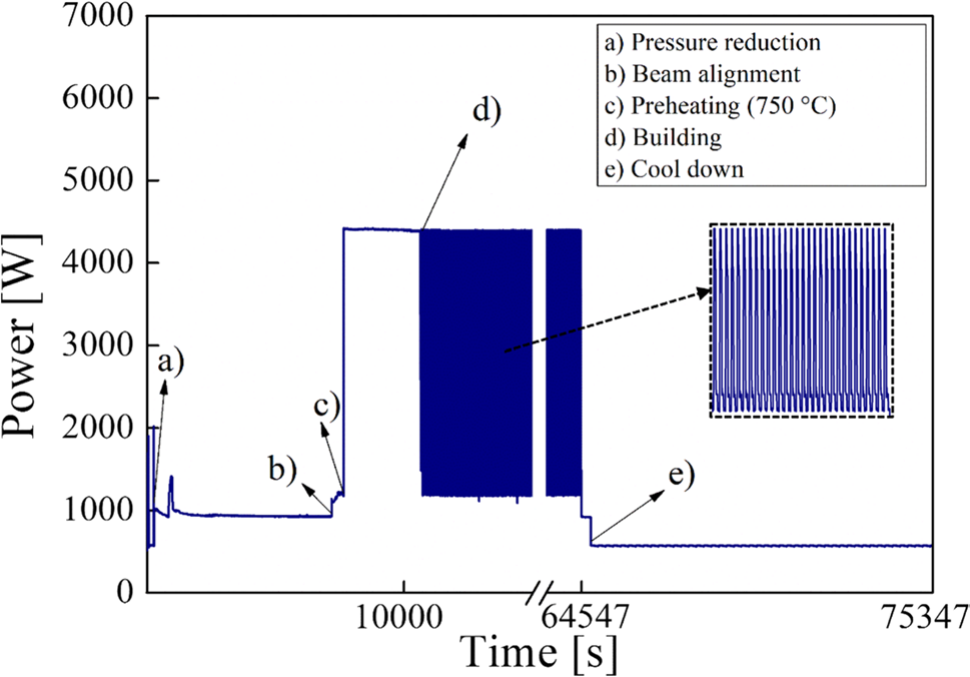
Power consumption during the EBM process

Figure 6 depicts the power trend of the whole EBM process to obtain the Ti6Al4V cylindrical samples into a single job for this study. Letters on the graph point out the beginning of the main subphases of the EBM process.

**Fig. 7:**
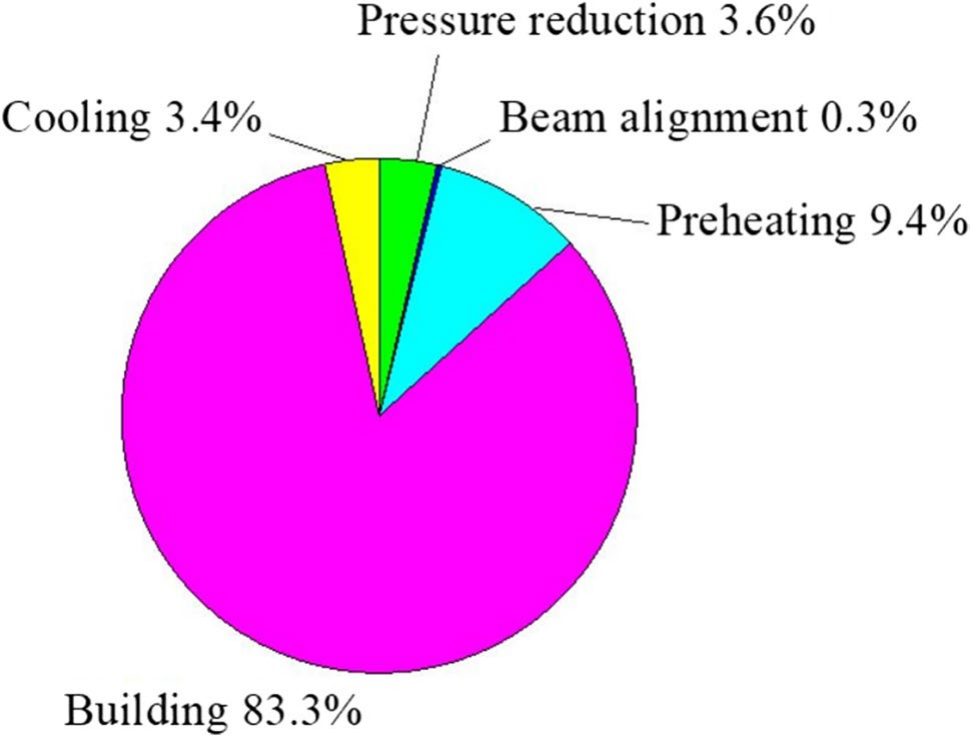
Energy consumption percentages of the subphases of the EBM process

**Fig. 8:**
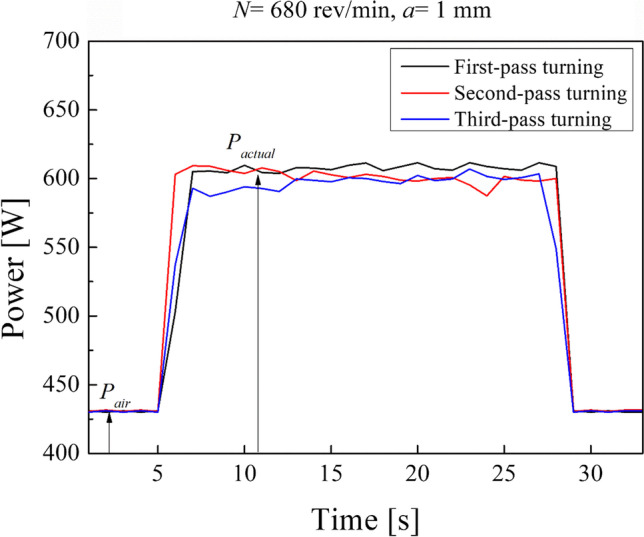
Power trend over time of the three turning passes on the sample number 5 (*N* = 680 rev/min, *a* = 1 mm)

As observed in Fig. 6, the maximum power consumption in the EBM process is during the preheating of the start plate, which takes about 50 min to reach a temperature of 750 °C. Once the desired vacuum level in the build chamber and the beam alignment are achieved, the start plate is preheated. Then, processing of individual layers occurs and is repeated until the final layer is processed. Thus, the samples are built layer by layer in three steps: raking across the plate while depositing the powder, preheating, and melting of the layer. Once the samples have been built, the power demand drops because the electron beam, the rake and start plate unit, and the vacuum pumps are no longer powered. Only the power consumption due to the machine electronics is observable in this subphase. Then, after the build chamber is left to cool, the samples are removed by the plate. Both the power trend during the EBM process and the power values obtained during the single subphases and observable in Fig. 8 agree with the literature [31]. Table 3 contains the power average and time of the main subphases of the EBM process. The preheating requires the maximum average power while the building subphase lasts the longest, as expected. By calculating the energy consumption for each subphase as the product between the average power and the time, it can be observed that the building is the most energy-consuming subphase while the beam alignment is the least one. For better comprehension, a pie chart showing the energy consumption percentages of the main subphases of the EBM process has been realized in Fig. 7. Thus, the maximum percentage regards the building subphase, which is 83.3%, while the minimum percentage regards the beam alignment which is 0.3%.

**Table 3:** Average power, time, and energy consumption of the subphases of the EBM process

Subphase	Average power (W)	Time (s)	Energy consumption (MJ)	Energy consumption (%)
Pressure reduction	930	6883	6.4	3.6
Beam alignment	1170	469	0.5	0.3
Preheating	4400	3830	16.9	9.4
Building	2791	53,365	148.9	83.3
Cooling	570	10,800	6.1	3.4

Specific energy consumption (SEC) was adopted to evaluate the energy efficiency of the EBM process. It is the energy consumption to fabricate the mass unit by EBM and in our study is expressed as

3 $${\mathrm{SEC}}_{\mathrm{EBM}}={E}_{\mathrm{build}}/(n*{m}_{\mathrm{build}})$$

where [32] ${E}_{\mathrm{build}}$ (MJ) is the electrical energy consumption during the building of a single sample, and the product between ${m}_{\mathrm{build}}$ (kg), that is the weight of a manufactured sample, and $n$, that is the number of samples manufactured into the same job that in this study is equal to 9. As mentioned before, in this study, all the samples were manufactured into a single job and have a mass unit of about 330 g. Thus, by dividing the energy consumption for building the samples, ${E}_{\mathrm{build}}$, of 148.9 MJ (Table 3) for the product between the number of samples manufactured into the same job and the mass unit, a ${\mathrm{SEC}}_{\mathrm{EBM}}$ of 50.13 MJ/kg is obtained. Thus, an energy consumption of 50 MJ is required to build a kilogram of Ti6Al4V by EBM. This result agrees with the literature by taking into account that energy efficiency increases as a consequence of the SEC decrease due to the increase of the manufactured samples in the same job has been increased [14, 15].

Figure 8 depicts the trend of power upon the time during the turning process of sample number 5. It can be seen that the three curves representing the three turning passes on sample number 5 are nearly overlapped. In the beginning, all the power curves increase linearly with time while the cutting tool starts to come in contact with the sample. This is due to the fact that the cutting force increases as the cutting tool progressively approaches the workpiece, assuming that the specific cutting pressure is always the same. Then, while the tool is advanced into the work causing the cutting action, both the specific pressure and the cutting force are almost constant, and in fact, a power oscillating around 600 W can be observed during this stage of the process. Finally, at the end, the power curve decreases linearly because the cutting force decreases while the tool moves away from the workpiece. In Fig. 8, it can be seen that the curve of the first pass is lightly shifted upmost than the curve of the second pass, this can be attributed to the different microstructure (made of thinner lamellae, who leads to a higher hardness) observed in the outer region of the samples.

The results regarding power and energy consumption in the turning process are summarized in Table 4.

**Table 4:** Power and energy consumption in the turning process

No	*N* (rev/min)	*a* (mm)	${n}_{c}$	Total depth of cut (mm)	${V}_{c}$(m/min)	MRR (mm^3^/s)	${P}_{\mathrm{actual}}$(W)	${P}_{\mathrm{air}}$(W)	${P}_{\mathrm{cut}}$(W)	SCE (J/ mm^3^)	${T}_{m}$(s)	${E}_{m}$(kJ)	${E}_{nm}$(kJ)	${E}_{\mathrm{TOT}}$(J)
1	440	0.5	6	3	42	77	453	400	53	0.77	30	82	10	92
2	440	1	3	3	42	154	520	400	120	0.78	30	47	4	51
3	440	1.5	2	3	42	231	595	400	195	0.84	30	36	2	38
4	680	0.5	6	3	64	117	523	430	93	0.79	20	63	11	74
5	680	1	3	3	64	235	603	430	173	0.74	20	36	4	40
6	680	1.5	2	3	64	352	750	430	320	0.9	20	30	2	32
7	1120	0.5	6	3	106	194	858	497	361	1.86	12	62	12	74
8	1120	1	3	3	106	389	1061	497	564	1.44	12	48	5	43
9	1120	1.5	2	3	106	583	1183	497	686	1.18	12	29	2	31

${P}_{\mathrm{cut}}$ has been calculated according to the work of Younas et al. [33]: they acquired power consumption when the tool was not engaged in cutting the material while all the components were electrically energized, known as air cut power ${P}_{\mathrm{air}}$, and when the material is removed by the work–tool engagement, known as actual power ${P}_{\mathrm{actual}}$, to calculate the specific cutting energy (SCE) [34]. This cycle was repeated for each combination of process conditions. The cut power, ${P}_{\mathrm{cut}},$ is calculated as the difference between the actual power and the air cut power:

4 $${P}_{\mathrm{cut}}= {P}_{\mathrm{actual}}- {P}_{\mathrm{air}}$$

Thus, the specific cutting energy (SCE) (J/mm^3^) was calculated by the normalization of the air cut power ${P}_{\mathrm{air}}$ over the MRR:

5 $$\mathrm{SCE}={P}_{\mathrm{cut}}/\mathrm{MRR}$$

The workpiece length ${l}_{m}$ was of 50 mm for all the samples and for each combination of process parameters. The machining time ${T}_{m}$ (min) was calculated as follows:

6 $${T}_{m}= {l}_{m}/l$$

where $l$ is the cutting length per minute calculated from the feed rate and the spindle speed:

7 $$l=f*N$$

The machining time in seconds is obtained by multiplying the machining time ${T}_{m}$ in minutes with 60. Instead, the non-machining time, ${T}_{nm}$, is the time required for the cutting tool to move from the compensation position to the front cutting position in second [35, 36]. It represents the time between one pass and the next one and it is 5 s. Thus, the total energy consumption in the turning process involves the energy consumption for machining and the energy consumption for non-machining. The energy consumption for machining was calculated as follows:

8 $${E}_{m}={P}_{\mathrm{actual}}*{T}_{m}*{n}_{c}$$

where ${n}_{c}$ is the number of cutting passes to turn 3 mm of material, according to the specific combination of process parameters, while the energy consumption for non-machining was calculated as follows:

9 $${E}_{nm}={T}_{nm}*{P}_{\mathrm{air}}*({n}_{c}-1)$$

Thus, the total energy consumption in the turning process was obtained for all the samples as

10 $${E}_{\mathrm{TOT}}={E}_{m}+{E}_{nm}$$

where ${E}_{m}$ and ${E}_{nm}$ were calculated as expressed by the Eqs. 8 and 9.

The results of the roughness measurements are summarised in Table 5.

**Table 5:** Results of the roughness measurements

Sample	${\mathrm{Ra}}_{//} \left(\mathrm{\mu m}\right])$ as built	${\mathrm{Ra}}_{\perp } \left(\mathrm{\mu m}\right])$ as built	${\mathrm{Ra}}_{//} (\mu \mathrm{m})$ machined	${\mathrm{Ra}}_{\perp } (\mathrm{\mu m})$ machined	Reduction %${\mathrm{Ra}}_{//}$	Reduction %${\mathrm{Ra}}_{\perp }$
1	32.05	25.55	4.95	0.48	85	98
2	29.98	29.62	4.70	0.82	84	97
3	29.33	34.00	5.38	0.54	82	98
4	30.63	15.66	5.27	0.54	83	96
5	37.95	27.81	5.40	0.69	86	97
6	39.36	25.98	6.28	0.44	84	98
7	38.16	28.45	1.51	0.26	96	99
8	27.09	24.03	6.04	0.44	78	98
9	31.06	30.90	10.14	2.3	67	92

The roughness profile measurements carried out before the turning process along the parallel direction to the building one, indicated as ${\mathrm{Ra}}_{//}$, showed a range of the Ra value going from 27.09 to 37.95 µm. Similarly, the range of Ra values along the perpendicular direction, indicated as ${\mathrm{Ra}}_{\perp }$, to the building one goes from 15.66 to 34.00 µm. These high values are well in agreement with the literature [37] but are also not adequate for high performances applications where EBMed titanium parts are usually used [38], so this evidence corroborates the needing for post-building surface treatments. As expected, roughness decreased after the turning process. The minimum value of ${\mathrm{Ra}}_{//}$ obtained was 1.51 µm, and its maximum value was 10.14 µm. Instead, the minimum value of ${\mathrm{Ra}}_{\perp }$ obtained after the turning was 0.26 µm while its maximum value was 2.3 µm. Hence, higher roughness values are obtained along the direction parallel to the axis of the cylindrical samples than roughness values obtained along the direction perpendicular, as expected as a result of the turning process.

## Discussion

**Fig. 9:**
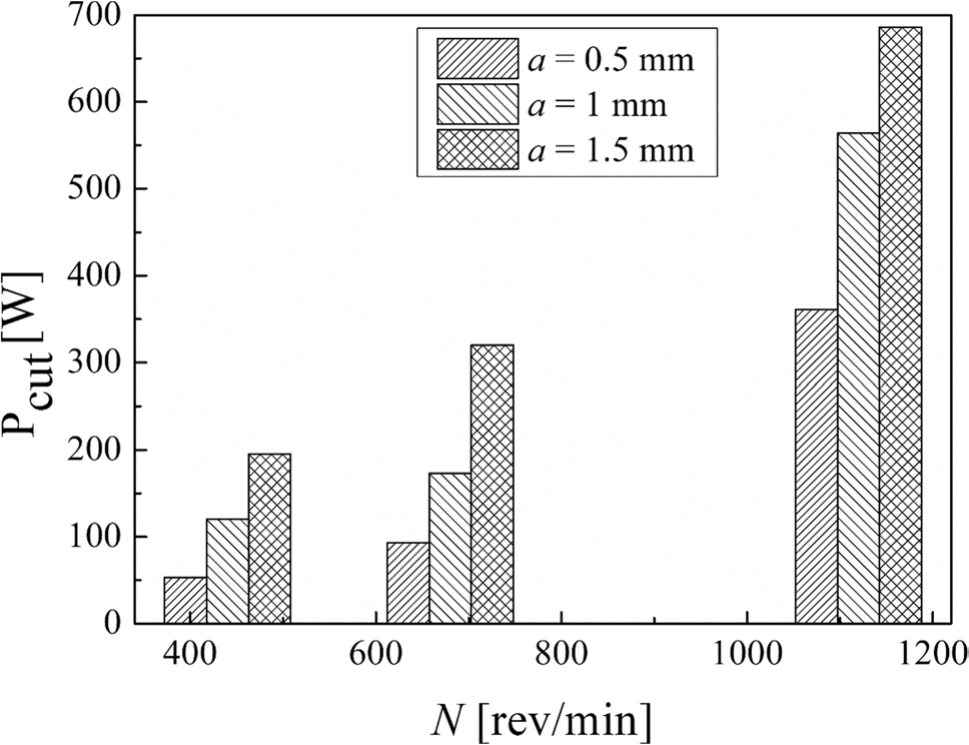
Cut power against the spindle speed for each depth of cut adopted

In Fig. 9, it is reported the cut power against the spindle speed according to the results obtained in this experimental campaign.

**Fig. 10:**
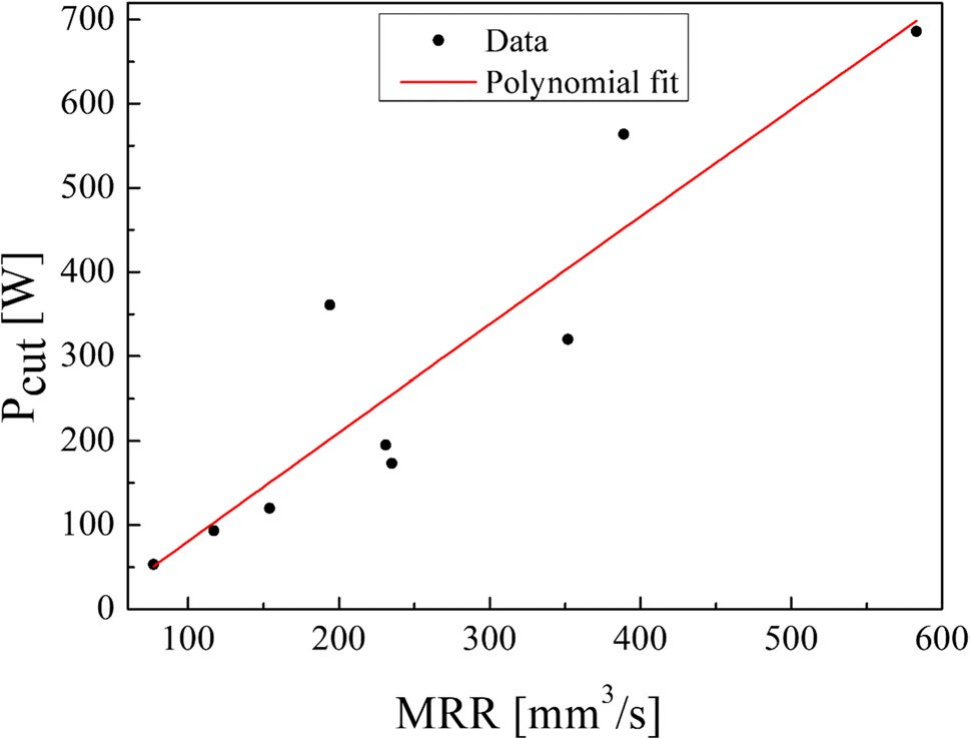
Linear relationship between the cut power and the MRR

**Fig. 11:**
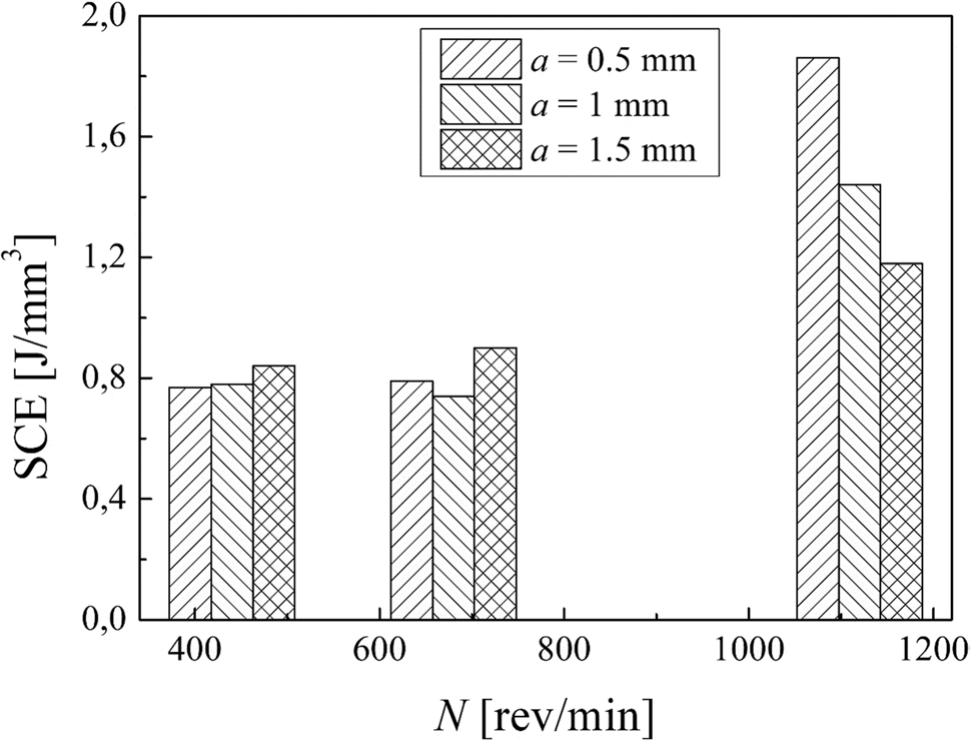
Specific cutting energy against the spindle speed for each depth of cut adopted

Indeed, by plotting the cut power values measured by the analyzer against the MRR (Fig. 10), a linear relationship is observed. Thus, cut power increases by increasing the MRR. Concerning the specific cutting energy (SCE), Eq. 5, a minimum value of 0.77 J/mm^3^ and a maximum value of 1.18 J/mm^3^ were found in our experimental campaign (Table 4). By plotting the data obtained (Fig. 11), no clear trend can be seen in SCE against the spindle speed. It can be observed that by choosing a spindle speed of 440 rev/min and 680 rev/min, a SCE less than 1 J/mm^3^ is obtained while a SCE greater than 1 J/mm^3^ is obtained with a spindle speed of 1120 rev/min. The results obtained show also that the air cut power, ${P}_{\mathrm{air}}$, increases linearly with the spindle speed (Table 4). Thus, the linear dependence of the power consumption by the spindle speed exists also when the cutting tool is not engaged in cutting the material while all the components are electrically energized.

**Fig. 12:**
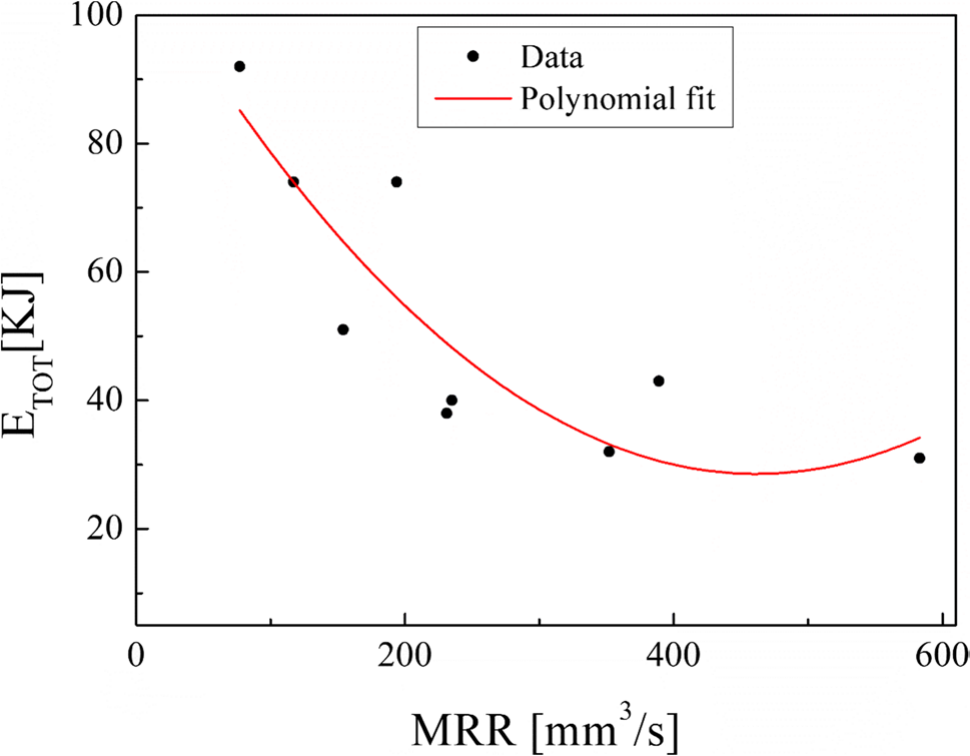
Total energy consumption in turning against the MRR

**Fig. 13:**
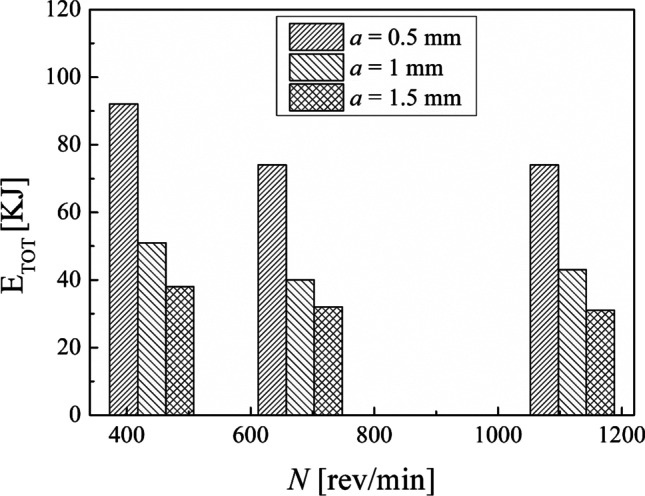
Total energy consumption in turning against the spindle speed

The results obtained in terms of total energy consumption in the turning (Eq. 10) show that the maximum total energy consumption is associated with the minimum MRR adopted while the minimum total energy consumption for turning is associated with the maximum MRR of the experimental design adopted in this study. Figure 12 shows clearly that the total energy consumption decreases by increasing the MRR. The depth of cut chosen seems to be highly decisive on the total energy consumption in the turning process, as observable in Fig. 13. In fact, the total energy consumption oscillates around the same value by fixing the depth of cut and by varying the spindle speed. This is explained by the fact that by decreasing the depth of cut, the number of turning passes increases to turn the same total depth of cut (3 mm in this study).

The energy consumption analysis in the EBM process highlighted that the maximum power consumption is during the preheating of the start plat to reach 750 °C. As expected, the maximum energy consumption percentage regarded the subphase of building the samples while the minimum percentage regarded the beam alignment (Fig. 7). The specific energy consumption (SCE) index was adopted to evaluate the energy consumption to manufacture a kilogram of Ti6Al4V samples. The calculation of SEC showed that energy consumption of 50 MJ is required to build a kilogram of Ti6Al4V by EBM, agreeing with the literature results [14].

Concerning the turning process, the cut power was calculated by means of the actual power and the air cut power (Eq. 4) and then the specific cutting energy (SCE) was calculated (Eq. 5) as well as the cutting time, the cutting length, and the total energy consumption were obtained for each combination of process parameters (Eqs. 6, 7, and 10).

The results showed that the machining time decreases by increasing the spindle speed (Table 4). Moreover, the MRR increases while the cutting speed increases (Eq. 2) as well as MRR increases, while the depth of cut increases when fixing the cutting speed. Both cut power and cutting speed depend linearly on the spindle speed. Instead, no clear trend has been observed in specific cutting energy against the spindle speed. Also, the results obtained show that the air cut power depends linearly on the spindle speed. Thus, the linear dependence of the power consumption by the spindle speed exists also when the cutting tool is not engaged in cutting the material while all the subsystems of the lathe are electrically energized.

The power records show that when the cutting tool comes in contact with the surface of the as-built sample, greater cutting forces are involved due to higher roughness than the following ones. It results in higher power consumption during the first turning pass. Thus, the first turning pass can be considered a roughing operation that leads to minimizing the undulations of the surface finishing of the samples manufactured by EBM.

The results of the calculation in the turning process (Table 4) demonstrated that the total energy consumption decreases by increasing MRR. Conversely, the total energy consumption oscillates around the same value by fixing the depth of cut and by varying the spindle speed. This is since the number of turning passes increases by decreasing the depth of cut to turn the same total depth of cut of 3 mm. Also, by comparing the results obtained in terms of Ra percentage reduction (Table 5), it can be observed that the best result of both ${\mathrm{Ra}}_{//}$ and ${\mathrm{Ra}}_{\perp }$ corresponds to the first combination of the experimental design by reducing up to 85% and 98%, respectively. Instead, the worst result regards the ninth sample, for which ${\mathrm{Ra}}_{//}$ and ${\mathrm{Ra}}_{\perp }$ were reduced by only 67% and 92%, respectively. Hence, the best choices of process parameters of turning to reach a better surface roughness are a lower both spindle speed and depth of cut.

**Fig. 14:**
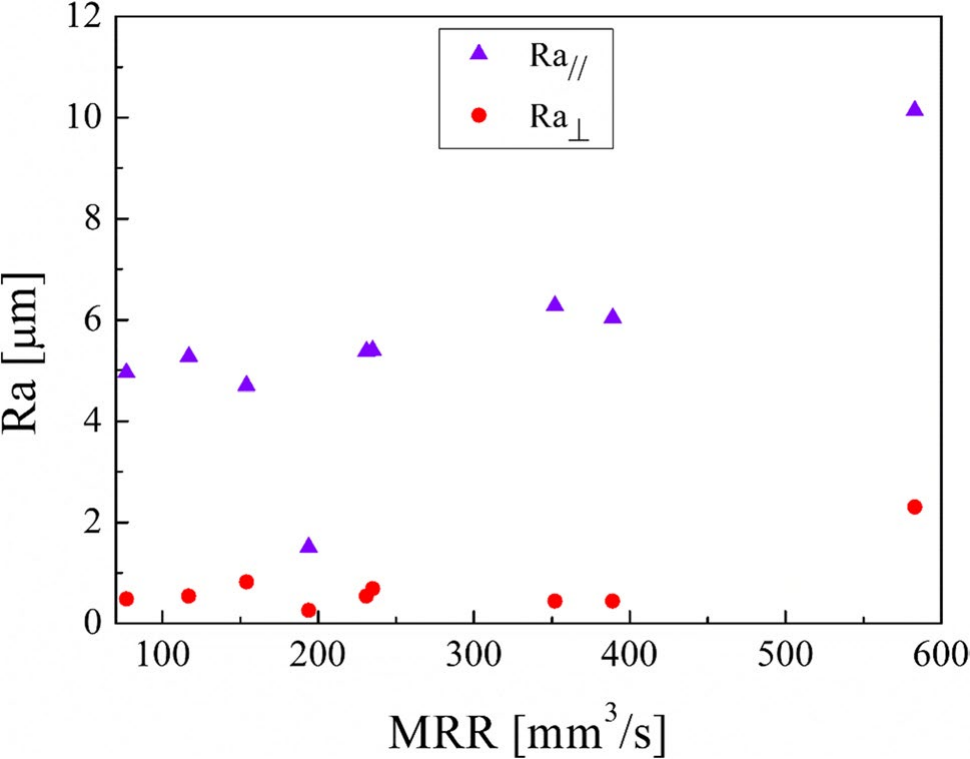
Roughness after the turning process along the principal directions of the cylindrical samples against the MRR

**Fig. 15:**
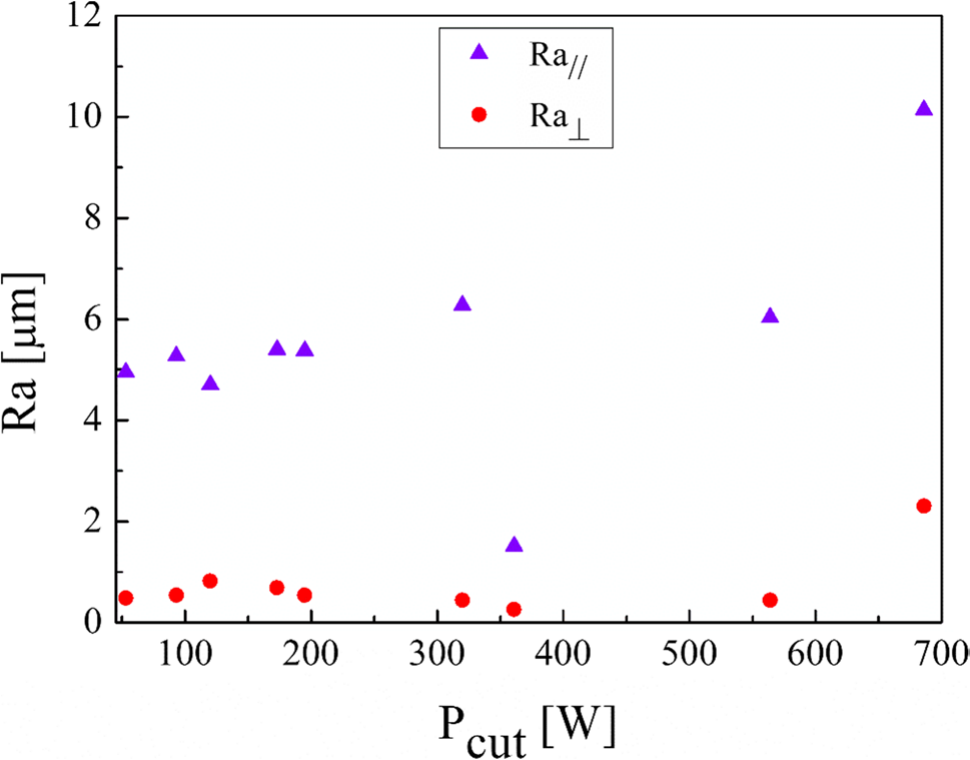
Roughness after the turning process along the principal directions to the axis of the cylindrical samples against the cut power

By jointly investigating the roughness and the energy consumption to obtain EBM parts post-processed by turning to have good surface roughness by minimizing the energy consumption, the following diagrams have been realized. First of all, the MRR can be considered an efficiency index, as it is the amount of material removed from the material per unit of time. Figure 14 shows that by increasing the MRR, roughness along the parallel direction increases whereas no clear trend is observed by plotting the roughness along the perpendicular direction to that of the building. As expected, the roughness shows the linear trend against the cut power as well as the cut power (Fig. 15).

**Fig. 16:**
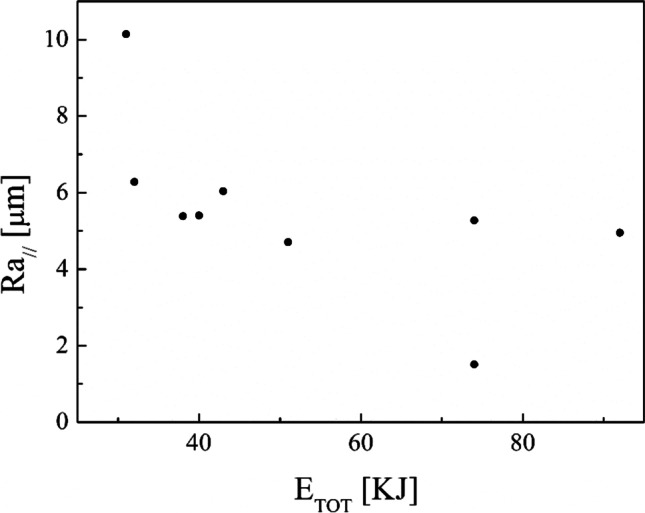
Roughness in parallel direction after the turning process against the total energy consumption in turning process

**Fig. 17:**
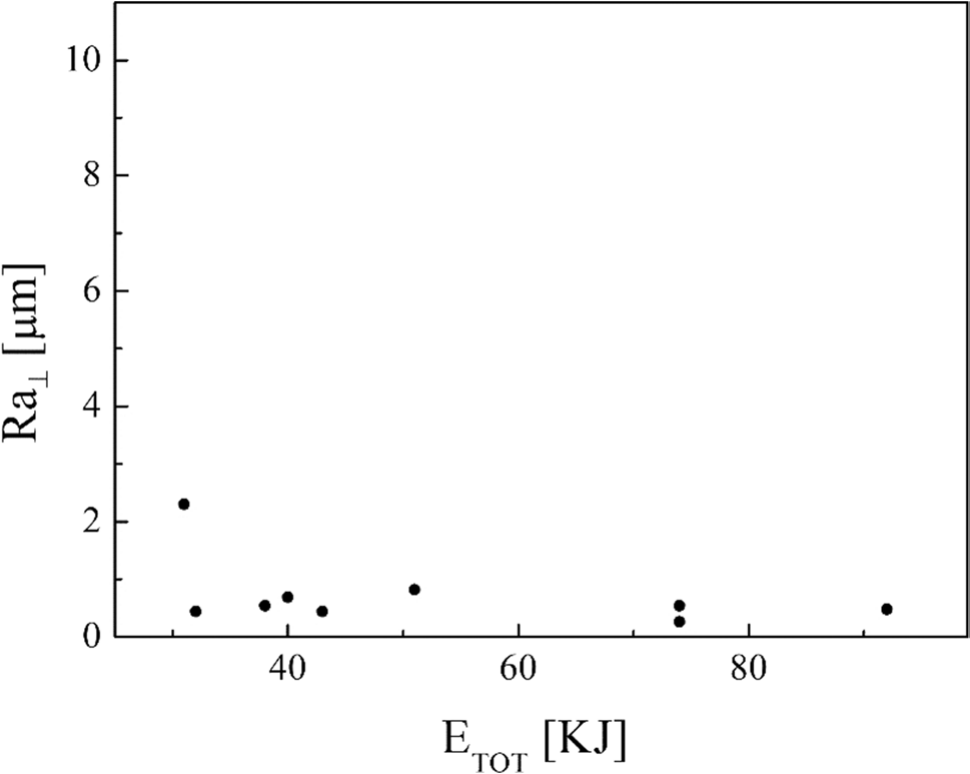
Roughness in perpendicular direction after the turning process against the total energy consumption in turning process

A joint analysis of the roughness (Table 5) and the total energy consumption in the turning process (Table 4) has been carried out and the following diagrams are the results. As can be seen in Fig. 16, the roughness along the parallel direction decreases from about 10 up to 2 µm by increasing the total energy consumption in the turning process. Instead, no clear trend can be seen in the roughness perpendicular direction by increasing the total energy consumption in the turning process (Fig. 17).

**Fig. 18:**
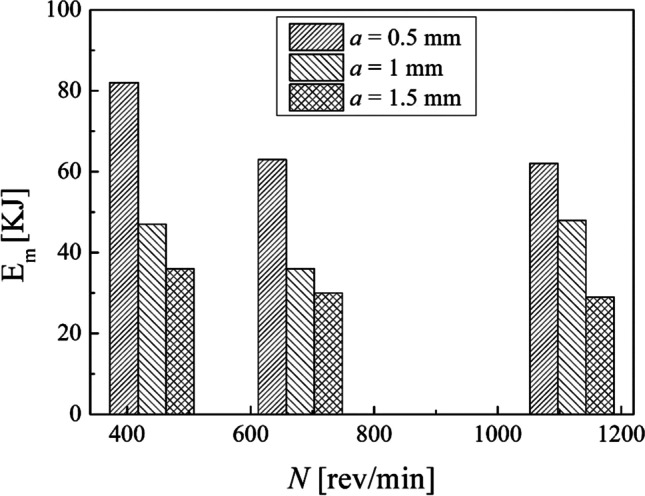
The total energy consumption during the machining time

**Fig. 19:**
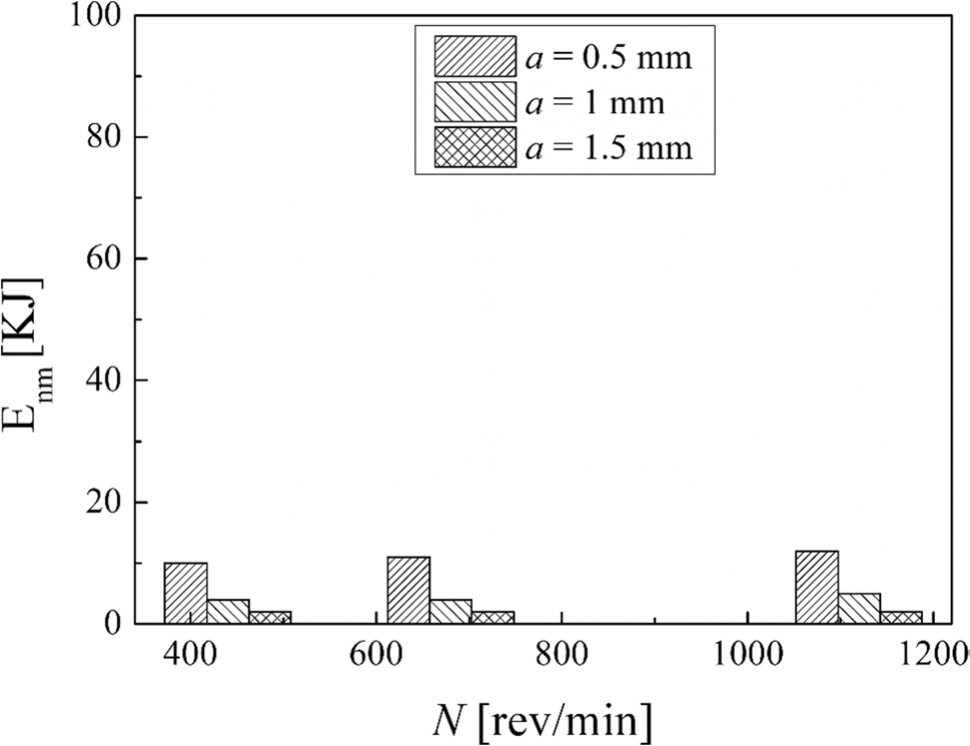
The total energy consumption during the non-machining time

Calculations by means of Eqs. 8, 9, and 10 were done to compare the results obtained by choosing different process conditions in turning. The results obtained in terms of the energy consumption during the machining time and the non-machining time are displayed in Figs. 18 and 19, respectively. It is observable that an increase in the spindle speed leads to a decrease in the energy consumption for either the machining time or the non-machining time. Moreover, by fixing the spindle speed, both the energy consumption for the machining time and the energy consumption for the non-machining time decrease while the depth of cut increases. Thus, from an energy-saving perspective, it is better to choose the highest spindle speed with the highest depth of cut.

Not only poor surface roughness and quality but also anisotropy and microstructural heterogeneity are drawbacks in metal additively manufactured parts. Typically, they affect the stiffness of the parts and the cutting forces in post-process machining. Pérez-Ruiz et al. [39, 40] investigated the effect of the laser powder bed fusion (LPBF) process parameters on both the cutting forces and the anisotropy in the peripheral milling of Inconel 718 parts. They found a correlation between the effect of the material anisotropy on the cutting forces and the tool position that could be explained through the crystallographic and grain morphology. In particular, a high energy density is associated with steeper melt pools and dense crystallographic textures, and they demonstrated that the Taylor factor enables a good prediction of the cutting force’s trend by varying the tool position.

Hojati et al. [41] investigated the machinability of Ti6Al4V parts in the micro-milling process by focusing on cutting forces and surface quality. The parts were manufactured by EBM technique and compared with the extruded ones. They observed no significant difference in the cutting force of both materials at chip thicknesses between 7.4 and 37.3 μm, despite the higher hardness of the EBM Ti6Al4V compared to the extruded Ti6Al4V. However, the cutting forces of EBM parts were less than those of extruded parts at minimal chip thickness (7.4 μm) which could be attributed to a dominating thermal softening effect at higher uncut chip thicknesses. In addition, a high deformation rate or strain rate through increasing the cutting speed at constant chip thickness has not resulted in higher cutting forces for both EBM and extruded Ti6Al4V. Hence, additively manufactured and conventional parts had the same material response at different strain rates.

## Conclusions

This experimental work shows the possibility of obtaining good surface finishing of Ti6Al4V parts manufactured by electron beam melting and then post-processed by turning in an energy-saving perspective. The results above discussed allow the enucleation the following points:

1. The automatic mode of the EBM machine led to a feasible and acceptable printing of nine Ti6Al4V cylindrical samples into a single job.
2. As a result of the EBM process, all the samples showed a high surface roughness. In particular, Ra ranging from 27.09 to 37.95 µm in the parallel direction to the building one and Ra ranging from 15.66 to 34.00 µm along the perpendicular one were obtained.
3. As a result of all the turning process conditions adopted in this study, the surface finishing of the samples has been improved to meet industrial quality requirements. In particular, higher roughness values are obtained in parallel direction to the axis of the cylindrical samples than those obtained along the direction perpendicular, as a technological signature of the turning process.
4. The lowest spindle speed (440 rev/min) and lowest depth of cut (0.5 mm) of the experimental campaign led to the best results in terms of surface roughness along both the principal directions of the cylindrical samples.
5. The building of the samples is the most energy-consuming subphase of the EBM process while the beam alignment is the least one; they require 85.7% and 0.3% in energy consumption of the process, respectively.
6. Specific energy consumption, ${\mathrm{SEC}}_{\mathrm{EBM}}$, for building nine Ti6Al4V cylindrical samples by EBM into a single job is 50.13 MJ/kg.
7. A linear relationship was observed between the cut power and the MRR as well as between the air cut power and the spindle speed.
8. It was observed that if the MRR, given by the input process parameters in turning, increases, the total energy consumption in the turning process decreases. In particular, results showed a high statistical correlation exists between the input MRR and the outputs energy consumption and roughness. Thus, the MRR can be considered as productivity index to predict both the sustainability and the good surface finishing of a post-machined part.
9. Higher spindle speeds lead to lower energy consumption for both the machining time and non-machining time.
10. By fixing the spindle speed, both the energy consumption for the machining time and the energy consumption for the non-machining time decrease by increasing the depth of cut. For this reason, both higher spindle speed and depth of cut in input are always associated with greater energy savings in output.

This work takes the first steps toward the assessment of the environmental impact of AM processes integrating the manufacturing stage with the needed post-processing. It clearly could be enhanced, and some directions for future developments can be recommended. For instance, in this study, the process conditions during the turning were varied for all the samples while those in the EBM process were fixed. In particular, the EBM machine worked in automatic mode. Further investigation may include the variation of the process parameters in the EBM process by fixing those in post-process machining. It would be interesting to investigate how to select the process parameters in the EBM process to obtain a better surface finishing of the parts while minimizing both the energy consumption and the cost of the AM process as well as the number of passes in the post-process machining.
